# Optimization and Characterization of Mesoporous Sulfonated Carbon Catalyst and Its Application in Modeling and Optimization of Acetin Production

**DOI:** 10.3390/molecules25225221

**Published:** 2020-11-10

**Authors:** Usman Idris Nda-Umar, Irmawati Ramli, Ernee Noryana Muhamad, Norsahida Azri, Yun Hin Taufiq-Yap

**Affiliations:** 1 Department of Chemistry, Faculty of Science, Universiti Putra Malaysia, Serdang 43400 UPM, Selangor, Malaysia; ernee@upm.edu.my (E.N.M.); norsahidaazri@gmail.com (N.A.); taufiq@upm.edu.my (Y.H.T.-Y.); 2 Department of Chemical Sciences, Federal Polytechnic, P.M.B. 55 Bida, Niger State, Nigeria; 3 Catalysis Science and Technology Research Centre (PutraCat), Faculty of Science, Universiti Putra Malaysia, Serdang 43400 UPM, Selangor, Malaysia; 4 Laboratory of Processing and Product Development, Institute of Plantation Studies, Universiti Putra Malaysia, Serdang 43400 UPM, Selangor, Malaysia

**Keywords:** mesoporous, carbon, sulfonation, acetin, modeling, optimization

## Abstract

In this study, an optimized mesoporous sulfonated carbon (OMSC) catalyst derived from palm kernel shell biomass was developed using template carbonization and subsequent sulfonation under different temperatures and time conditions. The OMSC catalyst was characterized using acid-base titration, elemental analysis, XRD, Raman, FTIR, XPS, TPD-NH_3_, TGA-DTA, SEM, and N_2_ adsorption–desorption analysis to reveal its properties. Results proved that the OMSC catalyst is mesoporous and amorphous in structure with improved textural, acidic, and thermal properties. Both FTIR and XPS confirmed the presence of -SO_3_H, -OH, and -COOH functional groups on the surface of the catalyst. The OMSC catalyst was found to be efficient in catalyzing glycerol conversion to acetin via an acetylation reaction with acetic acid within a short period of 3 h. Response surface methodology (RSM), based on a two-level, three-factor, face-centered central composite design, was used to optimize the reaction conditions. The results showed that the optimized temperature, glycerol-to-acetic acid mole ratio, and catalyst load were 126 °C, 1:10.4, and 0.45 g, respectively. Under these optimum conditions, 97% glycerol conversion (GC) and selectivities of 4.9, 27.8, and 66.5% monoacetin (MA), diacetin (DA), and triacetin (TA), respectively, were achieved and found to be close to the predicted values. Statistical analysis showed that the regression model, as well as the model terms, were significant with the predicted R^2^ in reasonable agreement with the adjusted R^2^ (<0.2). The OMSC catalyst maintained excellent performance in GC for the five reaction cycles. The selectivity to TA, the most valuable product, was not stable until the fourth cycle, attributable to the leaching of the acid sites.

## 1. Introduction

The use of biodiesel as a renewable green fuel to serve as an alternative to fossil fuels is challenged by the high cost of feedstock, which accounts for over 70% of the entire cost of production [1]. In recent times, efforts have been geared towards the use of low-priced feedstocks, namely inedible oils, waste cooking oils, and animal fats, to produce biodiesel [1,2]. Another alternative measure is to convert the byproduct, glycerol, to other more valuable products, thereby ameliorating the cost of biodiesel production and promoting a circular economy [3]. The worldwide production of glycerol is on the rise. It has been forecasted that by the end of the year 2020, 41.9 billion liters of glycerol will be available in the global market, and due to the surplus, it is expected that the price will fall dramatically [4]. Therefore, their conversion to other high-value products will improve the commercial viability of biodiesel.

Different catalytic reaction pathways have been reported in the conversion of glycerol. They include dehydration to acrolein, hydrogenolysis to 1,2 and 1,3-propanediol, oxidation to glyceric acid, dihydroxyacetone, tartronic acid, reforming to synthesis gas, etherification to ethers, acetalization to solketal and acetals, and acetylation to acetin, among others. Some of these products serve as intermediates in many reactions, while others are used as the end-product. Their applications cut across polymers, cosmetics, food, pharmaceuticals, tobacco, paints, fine chemical industries, and fuel additives [5,6,7].

The trend of the above catalytic reactions is fast changing from the use of homogeneous catalysts due to environmental concerns, difficulty in its separation, corrosive effects, and nonrecyclability to more advantageous heterogeneous solid acids catalysts [3,6,8].

**Scheme 1:**
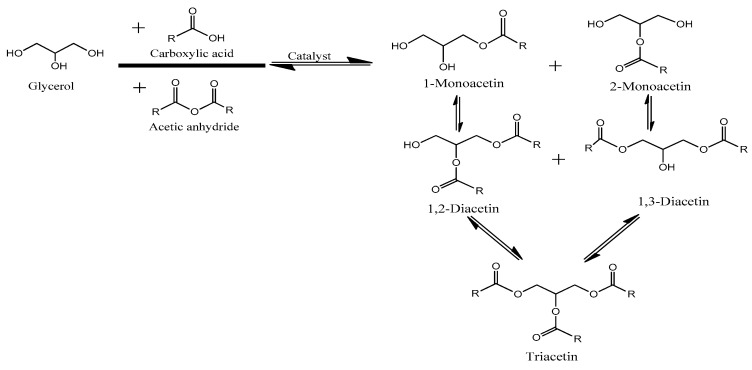
Glycerol acetylation with acetic acid or acetic anhydride to produce acetin. R = methyl group.

Many studies confirmed that acetylation of glycerol is an acid-catalyzed reaction leading to monoacetin, diacetin, and triacetin products, respectively [9,10,11]. It is a stepwise consecutive reaction, as indicated in Scheme 1. The three products have a versatile application. However, triacetin is considered the most valuable, yet its selectivity is limited despite the use of several types of heterogeneous catalysts such as a sulfonic acid resin (amberlyst-15, amberlyst-35, and amberlyst-36), heteropoly acid, zeolites, etc. [12,13,14]. The low selectivity has been attributed to the weakening acid strength of the catalysts, restricted access to the acidic sites, high molecular weight and active site ratios, narrow pores, presence of water, sequential acetylation of the hydroxyl groups, and low surface areas in some.

To improve triacetin selectivity, some researchers have deployed the use of acetic anhydride as the acetylating agent with 100% glycerol conversion and >90% triacetin selectivity, achieved within a short reaction time over some catalysts [15,16]. However, acetic anhydride is highly exothermic, more expensive, a potential substance for narcotic production, and is considered contraband in many countries [17,18]. Though many researchers have reported low selectivity with acetic acid, few have reported a high level of triacetin selectivity but under severe conditions. A good attempt was made by Rezayat and Ghaziaskar [19] when nearly 60% selectivity to triacetin and 40% to diacetin were obtained at a higher acetic-acid-to-glycerol molar ratio of 24:1 and high pressure of 200 bar over the amberlyst-15 catalyst. However, this molar ratio is not sustainable for industrial applications. Similarly, recent literature reported over 91% triacetin selectivity over the combined microporous molecular sieve HZSM-5 and mesoporous molecular sieve MCM-41 as a catalyst after 24 h [20]. This long hour of reaction is also not economical.

Therefore, research into the synthesis of catalysts to improve triacetin selectivity using acetic acid is still a topical research area. The potential of mesoporous materials in catalysis cannot be overemphasized because of their comparative advantage. They have a large surface area with tunable pore characteristics, various surface functionalities, and permit good diffusion. Carbonaceous materials can easily meet the mentioned characteristics, making them suitable for liquid phase applications, especially in catalysis. Our research group identified the use of biomass waste, palm kernel shell (PKS), as a catalytic material for glycerol acetylation. In the first phase, the potential of PKS as a carbon-based catalyst was reported in acetylation reaction and hence the need to optimize it.

The synthesized carbon-based catalysts resulting from different carbonization methods show good reaction activity; however, the catalyst prepared using template carbonization exhibited the highest selectivity of 58.9% to triacetin. The glycerol conversion was 97%, and the selectivity to monoacetin and triacetin were 5.8 and 32.2%, respectively, under the reaction conditions of atmospheric pressure, a temperature of 120 °C, a glycerol-to-acetic-acid mole ratio of 1:6, stirring at 450 rpm, and a reaction time of 3 h [21]. This result is promising when compared to earlier studies using acetic acid. The results of Okoye et al. [18], Goscianska and Mailakal [22], Kakasaheb et al. [23], and Marwan et al. [24] exhibited triacetin selectivity of 43, 22, 15, and 8.3% with very high glycerol conversion (>95%) over different catalysts, which were lower under relatively similar reaction conditions than our earlier study. However, the triacetin selectivity reported by Rezayat and Ghaziaskar [19] and Liu et al. [20] were much higher, as indicated earlier above, albeit under extreme conditions. Hence, the need to optimize our earlier study both in terms of catalyst synthesis and the conditions for acetylation reaction arises.

Several reports in the literature have also shown that different methods and conditions of carbonization and sulfonation processes are essential elements in the preparation of sulfonated catalysts [25,26,27]. Our earlier study identified the template carbonization of PKS at 800 °C, with 4 h being excellent in obtaining a mesoporous sulfonated catalyst [21]. Hence, in this study, only sulfonation temperature and time were varied to obtain an optimized mesoporous sulfonated carbon (OMSC) catalyst.

Similarly, the optimization of reaction parameters using a design of experiment (DOE) to improve triacetin selectivity has been scantly reported [3,11,28]. DOE has recently been employed in optimization studies, namely factorial design, Taguchi design, and response surface methodology (RSM), with each having advantages and otherwise. The factorial design gives more run for the least information as it does not predict the best factor levels for the desired outcome, while Taguchi gives the least run but only for individual variables and does not allow for interactive studies. The RSM gives a large number of runs but fewer than one factor at a time (OFAT); however, it gives more information and allows for both individual and interactive variable studies. It also uses the model to predict the desired outcome [11,29]. The aim of this study is to improve the mesoporous sulfonated carbon catalyst derived from PKS by varying the sulfonation conditions and to also optimize the acetin production with interest in improving triacetin, using the statistical and mathematical modeling tool, RSM, and, finally, to report the reusability of the catalyst.

## 2. Results and Discussion

### 2.1. Optimization of the Sulfonated Catalyst

**Figure 1:**
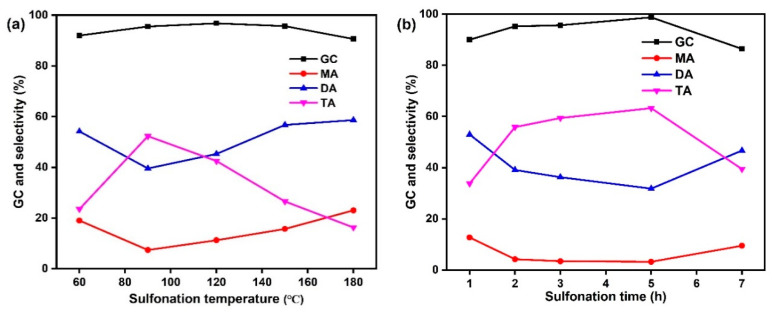
Effect of sulfonation conditions on the acetylation of glycerol with acetic acid catalyzed by the synthesized catalyst at 120 °C, a G/AA mole ratio of 1:6, a catalyst load of 0.5 g (10% G), and a reaction time of 3 h. (**a**) Sulfonation temperature (sulfonation time of 5 h) and (**b**) sulfonation time (sulfonation temperature of 90 °C).

Figure 1a shows that the glycerol conversion (GC) increases as the sulfonation temperature increases from 60 °C to 120 °C. It maintained high conversion until about 150 °C when it began to decline. However, the most valuable product of interest, triacetin (TA), increased when the sulfonation temperature increased from 60 °C to 90 °C, but after that, it sharply declined to favor the selectivity of DA. The observation may not be unconnected to the role of temperature in sulfonation, which is to introduce and stabilize the -SO_3_H acid groups into the polycyclic aromatic carbon structure of the catalyst and improve their acid densities [27,30]. The use of very low temperature leads to the formation of an unstable and easily hydrolyzed sulfonated catalyst with low acidity leading to low activity. High sulfonation temperature may cause an oxidation reaction between the concentrated sulfuric acid and the carbon material, leading to the partial destruction of the carbon structure with a reduced amount of -SO_3_H acid group substitution, reducing acidity and, consequently, lowering activity [26,30,31]. This can be observed in the trends of both the sulfonic and total acid densities in Table 1. The acidity increases to the peak and subsequently decreases as the sulfonation temperature increases. This study showed that a mild temperature of 90 °C is the optimal sulfonation temperature for the acetylation reaction, which also correlated with the highest acid density of the catalyst. Similar observations have been reported with similar and slightly varied optimum temperatures of 90 °C, 105 °C, 120 °C, and 135 °C, respectively, albeit with different biomass materials [25,26,27,30,32]. It is observed in Figure 1b that the GC and TA increase with the sulfonation time and peak at 5 h. The subsequent increase in the sulfonation time led to a sharp decline in both the GC and TA. This observation follows a similar trend with the acid densities presented in Table 1. This indicates that the introduction of an adequate amount of -SO_3_H and other acid groups is not relatively fast when compared to earlier studies that reported less than 1 to 3 h optimum sulfonation times for bamboo [27,31]. The difference may be attributed to the nature of the resultant carbon material derived from the various biomasses, carbonization temperature, and type of heating device applied. However, similar and higher sulfonation times ranging from 5 to 10 h have also been reported as optimal for corncob, willow catkins, oil palm trunk, sugarcane bagasse, and cacao shell materials [25,30,32,33].

A catalyst’s acid capacity is essential for the acetylation reaction [32,34]. The acidic contents of the synthesized carbon catalysts vary with different treatment conditions, as indicated in Table 1. The highest sulfonic and total acid densities were obtained with sulfonation treatment at 90 °C and a sulfonation time of 5 h. The value of total acid density is higher than the sulfonic acid density, implying the abundant presence of other acidic groups such as phenolic (-OH) and carboxyl (–c) in the sulfonated carbon catalysts [25,35]. The influence of these characteristics was seen in the acetylation reaction leading to the highest GC and TA selectivity as earlier reported above. Different methods were deployed in determining the acid densities to check their concurrence. The results showed that the values are not entirely the same but followed a similar trend. A similar finding has been reported by Lee [35].

### 2.2. Catalyst Characterization

Given our earlier report and the current findings, only the OMSC catalyst obtained at a sulfonation temperature of 90 °C for 5 h was characterized and further used for the optimization of acetin production using RSM.

#### Structural and Surface Characteristics of the OMSC Catalyst

**Figure 2:**
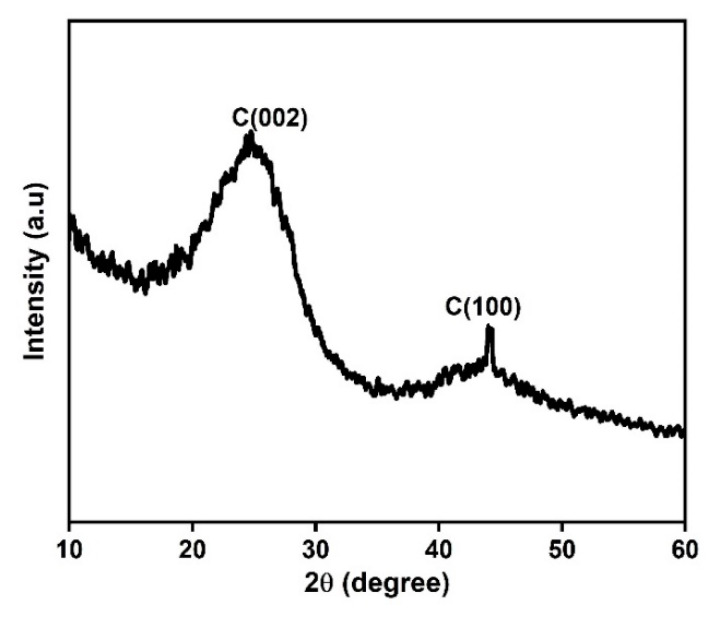
XRD diffractogram for the optimized mesoporous sulfonated carbon (OMSC) catalyst.

**Figure 3:**
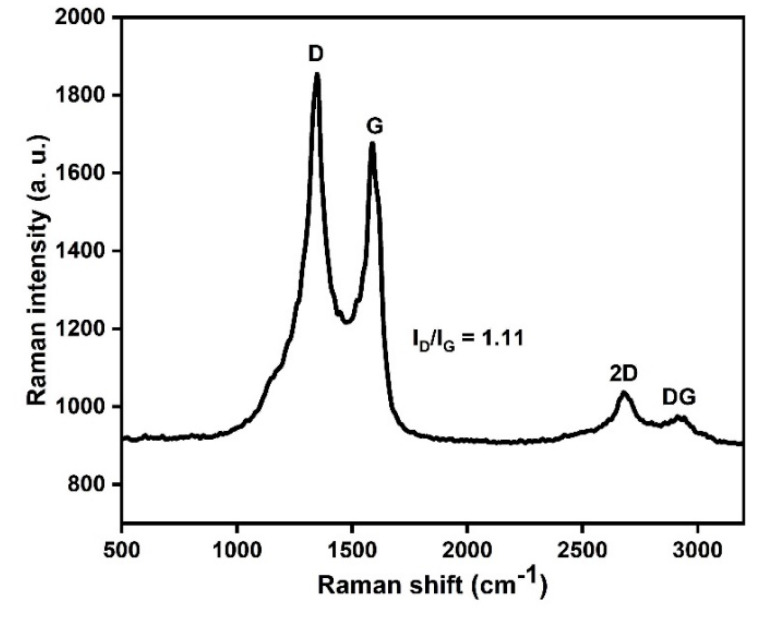
Raman spectra of the OMSC catalyst.

The XRD spectrum (Figure 2) of the OMSC catalyst maintained the two broad diffraction peaks at 2θ = 10–30° and 40–50°, corresponding to the amorphous carbon structure comprising polycyclic aromatic carbon sheets randomly oriented with some content of crystalline graphite [36]. The findings from the XRD result were further confirmed by the Raman spectrum (Figure 3), which exhibited two narrow bands in the first-order region (1000–2000 cm^−1^) with maxima at 1353 and 1589 cm^−1^, respectively. The first band corresponds to disorder (D-band) within the structure associated with breathing vibrations of sp^2^ carbon atoms in aromatic rings, while the second band is the graphite mode (G-band) associated with the in-plane bond stretching motion of sp^2^ carbon atoms [37,38]. There exist additional weak bands in the second-order region between 2500 and 3000 cm^−1^ associated with 2D and DG, which further confirms the amorphous carbon framework with high proportions of edge planes and structure defects [39,40]. The ratio of D-to-G bands (I_D_/I_G_) gives an idea of the degree of crystallinity. The value of 1.11 obtained for I_D_/I_G_ indicates that the degree of ordering in the carbon material is not too high, which is in line with amorphous materials. 

**Figure 4:**
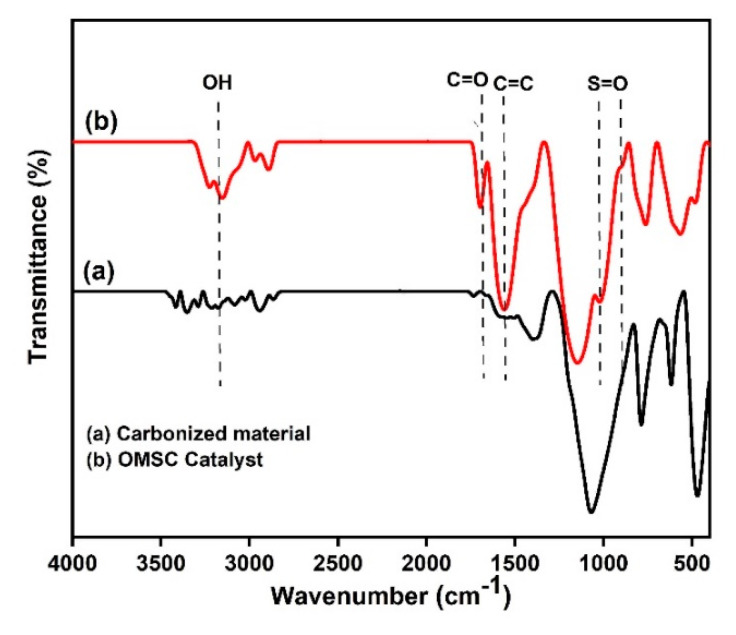
FTIR spectrum of (**a**) the carbonized material and (**b**) OMSC catalyst.

**Figure 5:**
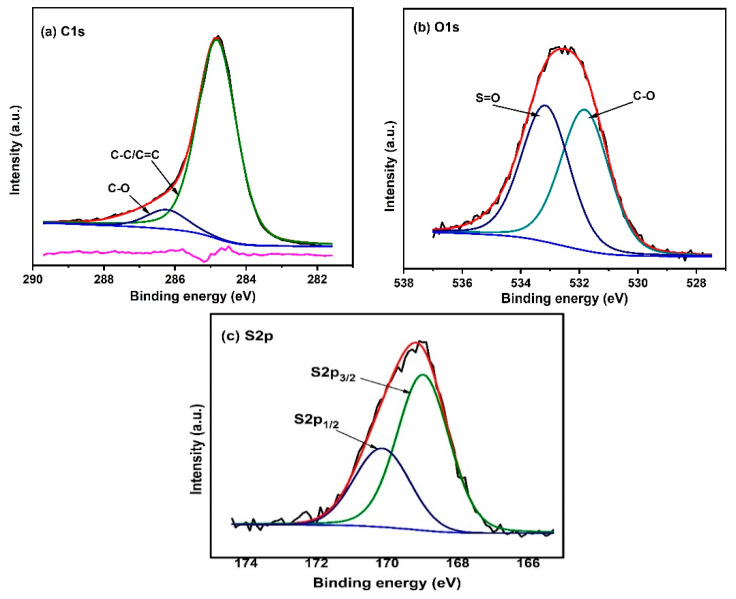
XPS spectra of (**a**) C 1s, (**b**) O 1s, and (**c**) S 2p of the OMSC catalyst.

The FTIR spectra of the OMSC catalyst is illustrated in Figure 4. It shows that the OH, C=O, and C=C stretching vibrations are intact and well pronounced at 3200, 1700, and 1600 cm^−1^, respectively. These functional groups indicate the presence of carboxyl groups attached to the aromatic carbons [35,41]. There is a shift in the S=O symmetric and asymmetric stretching vibrations observed at around 880 and 920 cm^−1^, respectively [25,35], in the sulfonated material (OMSC catalyst). The S=O stretching vibration is absent in the carbonized material (Figure 4b). The attachment of the sulfonic acid and other groups was further confirmed by the results of the XPS analysis illustrated in the narrow scans conducted in the C1s, O1s, and S2p regions, respectively, which are presented in Figure 5. The results in Figure 5a (C1s spectrum) show two deconvoluted peaks. The peak at 284.8 eV corresponds to the C-C/C=C bonds in the aromatic and aliphatic compounds, while that at 286.2 eV corresponds to the C-O bond in phenolic, alcoholic, and alkoxy compounds. Figure 5b shows the O1s spectrum with two deconvoluted peaks. The peaks at 531.8 and 533.2 eV correspond to C-O and S=O bonded to organic groups (i.e., aromatic rings and phenols). The S2p spectrum (Figure 5c) is deconvoluted into two peaks, S2p 3/2 and S2p 1/2, at 169.0 eV and 170.1 eV, respectively, which were assigned to sulfur in the SO_3_H groups, indicating successful sulfonation. The above functional groups were assigned in line with similar sulfonated carbon materials reported in the literature [37,42,43,44]. The observation of these functional groups and the data from FTIR confirmed the presence of a variety of acidic -SO_3_H, -COOH, and -OH groups. 

**Figure 6:**
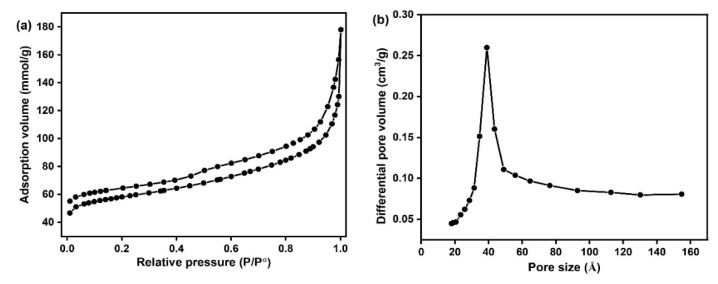
N_2_ physisorption isotherms (**a**) and pore size distributions (**b**) of the OMSC catalysts.

The evidence of the mesoporous catalyst can be seen in the Brunauer–Emmett–Teller (BET) physisorption isotherm and the Barrett–Joyner–Halenda (BJH) pore size distribution shown in Figure 6. The isotherm (Figure 6a) is of type IV with H3 hysteresis loops with mono- and multilayer adsorptions. The curves at relative pressure from >0.4 to 1.0 classified the catalyst as a mesoporous material. This was further confirmed by the pore size distribution (Figure 6b) centered between 30 and 50 Å [41,45]. The surface area and pore volume of the catalyst reduced after sulfonation optimization from 484 to 217 m^2^/g and from 0.327 to 0.164 cm^3^/g, respectively (Table 2), suggesting blockage of some pores arising from successful sulfonation process [22,43]. The increase in pore size of the catalyst may permit easy diffusion of the products out of the pores, which may be a contributing factor in the excellent performance of the catalyst in acetylation [34].

**Figure 7:**
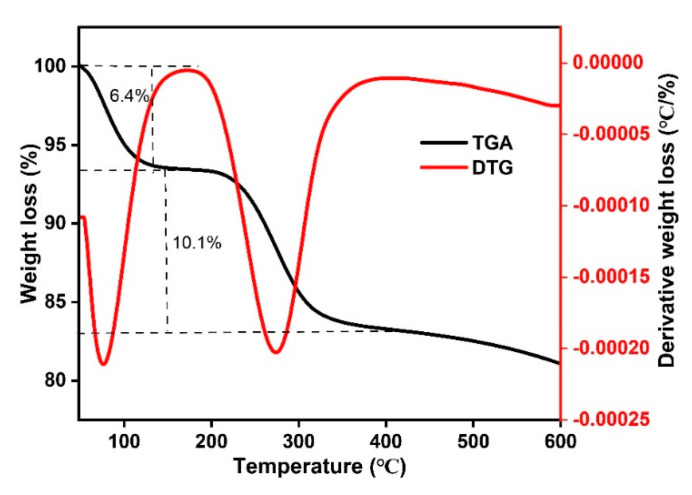
TGA and DTG curves of the OMSC catalyst.

The catalyst exhibited two major weight losses, as observed in Figure 7. The first weight loss, which was 6.4%, occurred below 100 °C and was attributed to the loss of moisture or water molecules, while the second weight loss of 10.1% occurred between 250 and 300 °C, attributable to the decomposition of –SO_3_H groups [43,46]. This observation indicates the high stability of the sulfonated catalyst and could be used at a temperature below 250 °C. These desorption peaks were confirmed in the DTG profile (Figure 7).

**Figure 8:**
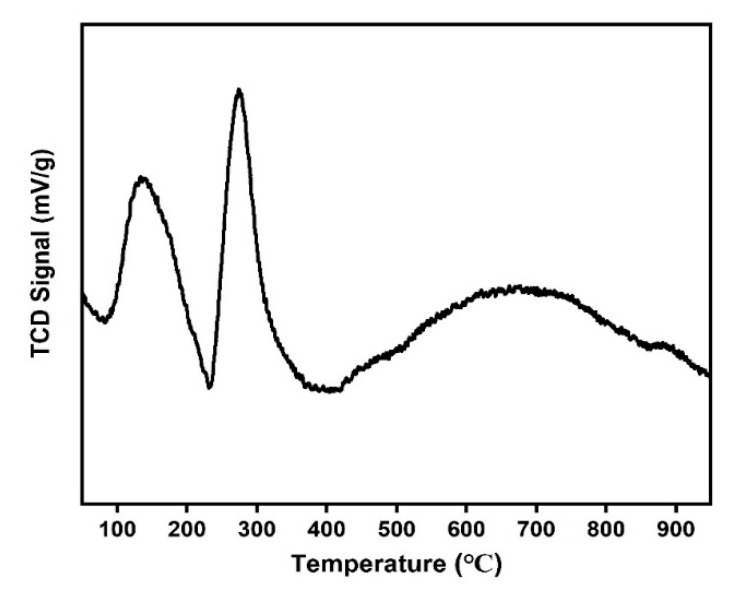
TPD-NH_3_ spectrum of the OMSC catalyst.

Similarly, the NH_3_-TPD profile of the OMSC catalyst (Figure 8) exhibited two prominent desorption peaks and a broad peak occurring between 100 and 200 °C, 250 and 350 °C, and >450 °C, respectively. The first desorption peak is attributed to the surface functional groups of -COOH, -OH, and water, while the second peak could be associated with the desorption of -SO_3_H groups. The third desorption peak is usually attributed to the CO surface commonly associated with phenolic and carbonyl/quinones functional groups; however, in this case, it may be associated with the decomposition of the carbon materials [47,48]. These observations correlated with the findings of the DTG and showed a similar but more distinct pattern when compared with the initial study. This observation could be attributed to the optimization process carried out. Meanwhile, the optimization led to the significant reduction in the surface area and the pore volume when compared with the carbonized material (Table 2) attributable to partial oxidation and destruction of the porous structure during the sulfonation process, leading to the attachment of more -SO_3_H groups on the carbon structure [27,49]. The pore diameter improved slightly, thereby making it suitable for reactants and products with a bulky structure, such as glycerol esters, to easily diffuse in and out of the pores [34,50]. The OMSC catalyst also exhibited high acid site density, as indicated by the NH_3_-TPD result (Table 2), and this will provide enough of an anchoring site for the reaction to occur. The above properties, which include high surface area, pore volume, and acid site density, must have provided easy access to the catalytic sites leading to the high catalytic performance.

**Figure 9:**
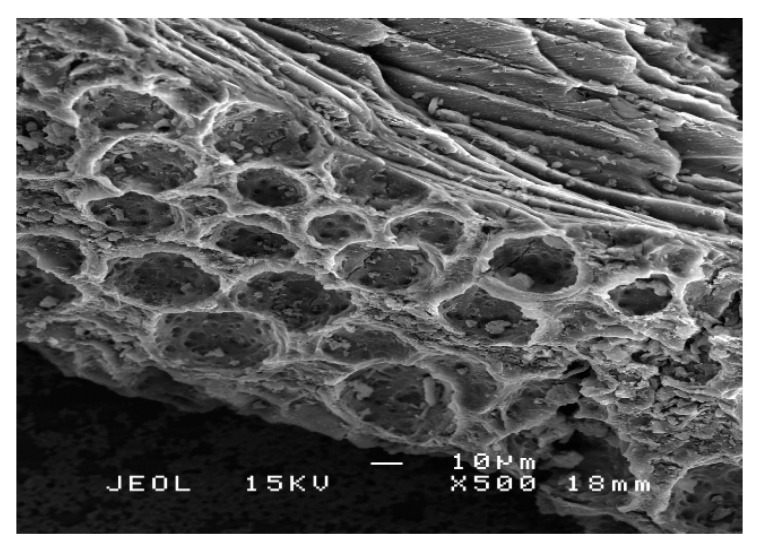
SEM micrograph of the OMSC catalyst.

The SEM micrograph of the mesoporous carbon catalyst (Figure 9) exhibited a regular and slightly rough network of porous structures with few fibrous-like ridged edges. The porous structure is composed of small to large cavities attributed to the use of the silica template and high temperature of carbonization. The removal of the template after carbonization must have also created numerous and large cavities. Similar reports have also shown large cavities in various carbon treated materials [51,52].

### 2.3. Modeling and Optimization of the Acetin Production

The actual experimental values obtained for the responses (glycerol conversion (GC), monoacetin (MA), diacetin (DA), and triacetin (TA)) are contained in the experimental design matrix in Table S1. The results were analyzed using the Design-Expert software 10.0.6.0 to obtain the statistical analysis of variance (ANOVA), regression model, and model fitness for all the responses to evaluate their suitability and accuracy. The ANOVA gives the statistical significance of the quadratic and the model terms (individual and interaction) based on Fisher’s F-test (F-value) and the probability value (*p*-value), where *p* < 0.05 is considered significant [53]. The regression models were obtained by multiple regression analysis. The final equation based on the significant factors was outlined. For model fitness, the coefficient of determination (R^2^) was used to indicate the agreement between the experimental and predicted values [54].

#### 2.3.1. Analysis of Variance (ANOVA), Regression Model, and Model Fitting for GC

Table S1 indicates that the minimum and the maximum values obtained for GC were 83.77 and 95.60%, respectively. The ANOVA result for the GC response is shown in Table S2. It showed the model has an F-value of 134.01 with *p* < 0.0001, which indicates a statistically significant model at the 95% confidence level. This means that there is only a 0.01% chance that an F-value this large could occur due to noise. The table also revealed that the linear terms (A, B, and C) of the model are significant with *p* < 0.05, while the interaction terms are not significant except the G/AA mole ratio and the catalyst interaction term (BC). All the quadratic terms are also significant, except that of the catalyst load (C^2^). The catalyst load (C) has the highest influence on the GC with an F-value of 569.65, followed by the G/AA mole ratio (B) with an F-value of 470.79. However, the influence of C is negative, indicating an antagonistic effect, whereas that of B is positive, indicating a synergistic effect.

Based on these results, the regression model in terms of the coded factors showing the relationship between the glycerol conversion (GC) and independent variables (temperature (A), G/AA mole ratio (B), and catalyst load (C)) after excluding the insignificant terms is represented by Equation (1).

(1) $$\mathrm{GC}=94.27+0.46A+3.75B-4.12C+1.68\mathrm{BC}+2.05A^{2}-2.66B^{2}$$

**Figure 10:**
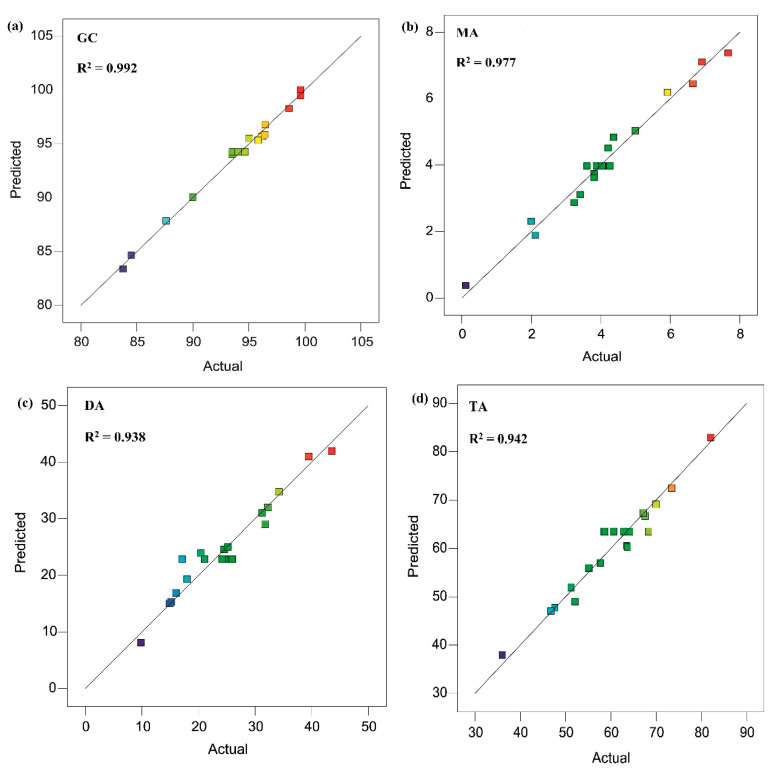
Parity plot for (**a**) glycerol conversion (GC) (**b**) monoacetin (MA) (**c**) diacetin (DA) and (**d**) triacetin (TA) selectivity.

The model fitness was evaluated. The coefficient of determination (R^2^) 0.9918 is close to unity, indicating that the fitted model could account for a 99.18% variation in the GC as obtained by the model. Zamzuri et al. reported that an R^2^ value of at least 0.75 is adequate and fitting to account for the variability of response. The “pred R^2^” value of 0.8995 is in reasonable agreement with the “adj R^2^” value of 0.9844 (the difference is <0.2) while the “adeq precision” value >4 shows an adequate signal-to-noise ratio, further indicating that the model fits the experimental data very well. This excellent correlation was further illustrated by the parity plot in Figure 10a with almost all the points of the predicted and actual GC close to the diagonal line [28].

#### 2.3.2. Analysis of Variance (ANOVA), Regression Model, and Model Fitting for MA Selectivity

From the experimental data, the MA obtained ranged from 0.11 to 7.67%, as shown in Table S1. The ANOVA result in Table S3 indicates that the model is significant at a 95% confidence level with an F-value of 46.45 and *p* < 0.0001. All the linear terms (A, B, and C) are significant with *p* < 0.05; however, the interaction and quadratic terms are not significant. Therefore, the final regression models, in terms of coded factors, relating to MA as a function of temperature, G/AA mole ratio, and catalyst load are given by Equation (2). The G/AA mole ratio (B) has a greater influence on the MA selectivity with an F-value of 320.45, followed by temperature (A) with an F-value of 71.64; however, the influence by both terms is antagonistic.

(2) MA = 3.98−0.98A−2.07B+0.45C

The R^2^ value (0.9766) is close to unity, indicating that the fitted model could account for 97.66% variation in the MA selectivity. The obtained “pred R^2^” value of 0.7852 agrees with the “adj R^2^” value of 0.9556, whereas the “adeq precision” value of 27.039 indicates an adequate signal for the model to be used to navigate the design space. The model fit the experimental data, as indicated by its correlation with the predicted data and illustrated in the parity plot in Figure 10b.

#### 2.3.3. Analysis of Variance (ANOVA), Regression Model, and Model Fitting for DA Selectivity

The DA selectivity obtained from the experimental data ranged from 9.84 to 43.51%, as indicated in the design matrix in Table S1. The model F-value was found to be 16.70 with *p* < 0.0001, which implies that the model is significant at the 95% confidence level (Table S4). Similarly, the model terms A, B, C, and BC are significant while others are not. The regression model, in terms of coded factors, is represented by Equation (3). The catalyst load (C) has the highest influence on the DA selectivity with an F-value of 108.03, followed by temperature (A) with an F-value of 18.46. The influence of both terms is also antagonistic.

(3) DA=22.85−4.06A−2.56B−9.82C−2.56BC

The R^2^ value (0.9376) implies that the fitted model could account for a 93.76% variation in the DA selectivity. The “pred R^2^” value of 0.7311 also agrees with the “adj R^2^” value of 0.8815 with a difference of <0.2. The “adeq precision” value of 16.019 indicates an adequate signal. The parity plot in Figure 10c illustrates that the points are close to the diagonal line, which also implies the correlation between the predicted and actual data.

#### 2.3.4. Analysis of Variance (ANOVA), Regression Model, and Model Fitting for TA Selectivity

The high selectivity of TA by the OMSC catalyst remains the main objective of this study. The experimental data in Table S1 indicate that TA selectivity ranged from 36.03 to 82.07%. Subjecting the experimental data to ANOVA indicate that the model is significant at the 95% confidence level with an F-value of 18.15 and *p* < 0.0001, respectively. The ANOVA result is shown in Table S5. It further reveals that A, B, BC, and C^2^ are the significant model terms. The regression model, in terms of coded factors, excluding the insignificant factors, is represented by Equation (4). The G/AA mole ratio (B) has the highest influence on TA selectivity with an F-value of 84.25, followed by temperature (A) with an F-value of 29.66. The influence of both terms is synergistic.

(4) $$\mathrm{TA}=63.51+5.95A+10.03B+6.30\mathrm{BC}-4.85C^{2}$$

The “lack of fit” F-value of 0.69 implies that it is not significant relative to the pure error, and the model is fit and desirable. The R^2^ value (0.9423) implies that the fitted model could account for a 94.23% variation in TA selectivity. The obtained “pred R^2^” value of 0.8148 is in agreement with the “adj R^2^” value of 0.8904 with a difference of <0.2. The “adeq precision” value of 18.411 indicates an adequate signal for the model to be used to navigate the design space. The coefficient of variation (CV) of 5.73% is less than 10%, thereby exhibiting an excellent correlation between the predicted and the actual TA selectivity, as illustrated in the parity plot in Figure 10d.

### 2.4. Effect of Variables and Their Interaction on GC and Selectivity to MA, DA, and TA

**Figure 11:**
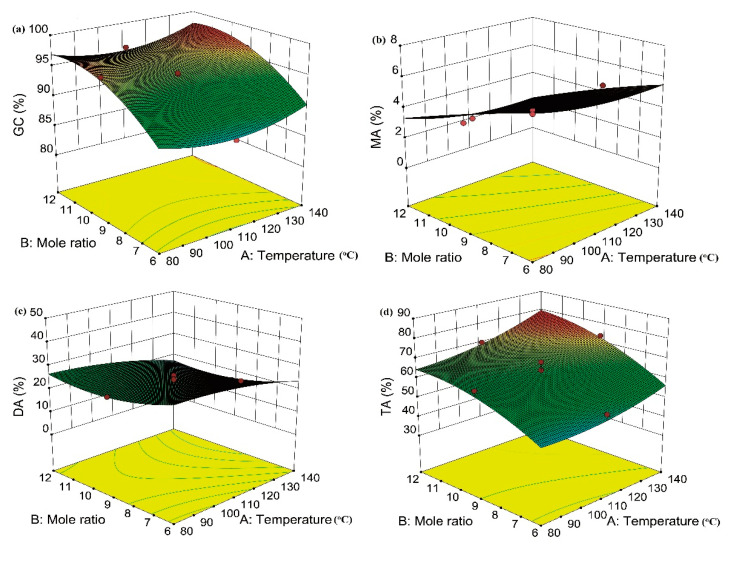
Surface response plots for (**a**) GC and selectivity to (**b**) MA, (**c**) DA, (**d**) TA as a function of the G/AA mole ratio, and temperature at a constant catalyst load (0.6 g) and reaction time (3 h).

Figure 11 exhibits the surface plots corresponding to the effect of the G/AA mole ratio and temperature on the GC and selectivity to MA, DA, and TA, respectively. Figure 11a indicates a concurrent increase in the G/AA mole ratio and the temperature led to an increase in GC. It was also observed that at a constant temperature, an increase in the G/AA mole ratio also increases GC correlating with the synergistic effect of the G/AA mole ratio term in Equation (5). This observation may be due to the presence of excess acetic acid, which drives the reversible reaction more towards the products [32,55]. The highest GC was achieved at the G/AA mole ratio of 1:9, and subsequently, no visible increment was observed. Similarly, an increase in GC was observed with increasing temperature at a constant G/AA mole ratio. This is also in line with the synergistic effect of temperature term in Equation (5). This is expected since glycerol acetylation is an endothermic reaction. Therefore, the reaction rate usually increases with temperature and enhances the diffusion of reactants and products in and out of active sites [14,55,56]. In contrast, a concurrent increase in G/AA mole ratio and temperature results in a decrease in both MA and DA, respectively (Figure 11b,c). This observation can be linked to their conversion to higher acetin and TA. Glycerol acetylation was a consecutive reaction where the formation of MA initially increased but later transformed into DA and subsequently TA [13,32]. It is evident from Figure 11d that TA increases with an increase in both the G/AA mole ratio and temperature. This may not be unconnected with increased acidity and improved diffusion efficiency of the reactants and products due to the high surface area [20].

**Figure 12:**
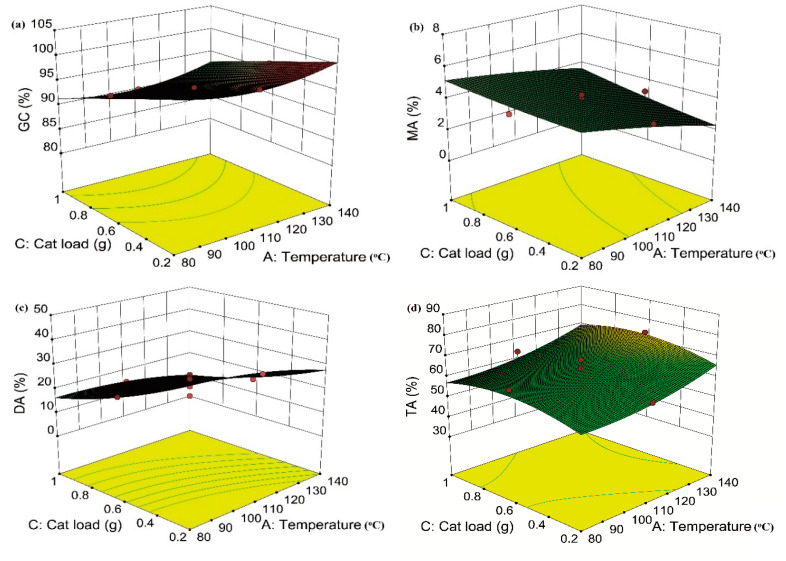
Surface response plots for (**a**) GC and selectivity to (**b**) MA, (**c**) DA, (**d**) TA as a function of the catalyst load, and temperature at a constant G/AA mole ratio (1:9) and reaction time (3 h).

Figure 12 shows the surface plots corresponding to the interaction effect of the temperature and the catalyst load on the responses (GC, MA, DA, and TA selectivity). Figure 12a indicates a high GC at high temperature and low catalyst load conditions. The figure also indicates that at a constant temperature, the GC decreases with an increase in catalyst load, which correlates with the antagonistic effect exhibited by the regression model (Equation (5)). This means that a low catalyst load has provided sufficient active sites for good GC in this study. Therefore, subsequent increments might have led to the agglomeration of catalyst particles, which may reduce the accessibility of the reactants to the catalyst’s active sites and hinder product transfer, thereby decreasing GC [55]. The catalyst load applied in this study appears not to have a significant effect on MA selectivity. Only a marginal increase was noticed (Figure 12b), but the DA selectivity decreased (Figure 11c) for the above similar reason. The interaction effect showed that MA selectivity was favored at low temperatures and any given catalyst load applied (Figure 12b), while the DA was favored at both low temperature and catalyst load (Figure 12c), thereby transforming it into TA [56]. High TA selectivity was favored at a catalyst load of 0.6 g and high temperatures (Figure 12d).

**Figure 13:**
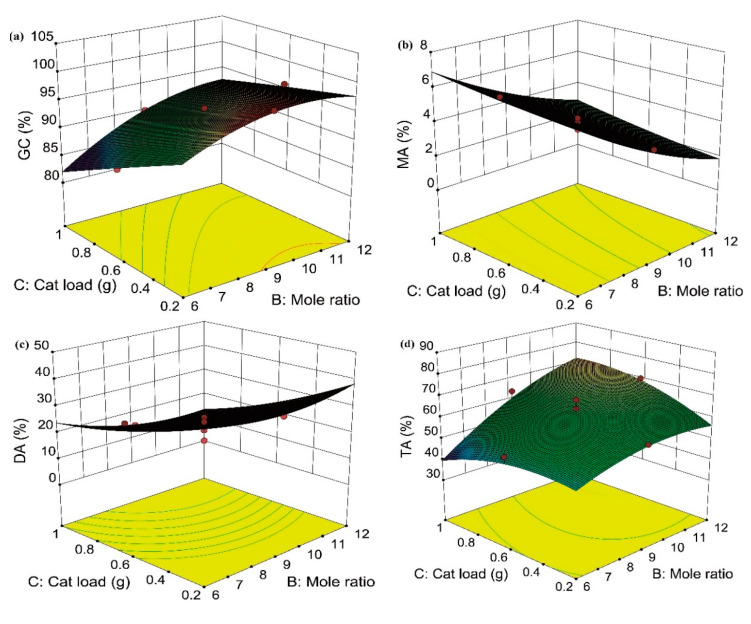
Surface response plots for (**a**) GC and selectivity to (**b**) MA, (**c**) DA, (**d**) TA as a function of the catalyst load, and G/AA mole ratio at a constant temperature (110 °C) and reaction time (3 h).

Figure 13a indicates that a low catalyst load with a simultaneous increase in the G/AA mole ratio improved GC. Figure 13b shows an improvement in MA when a low G/AA mole ratio was applied at any of the catalyst loads applied. The DA was favored at both a low catalyst load and G/AA mole ratio (Figure 13c). The TA selectivity was favored at a high catalyst load and high G/AA mole ratio (Figure 13d). The presence of excess reactant, as well as catalyst load, also improves the total number of active sites and acidity of the reaction medium, thereby shifting equilibrium more to the selectivity of TA [13,20]. 

### 2.5. Optimization

In this study, numerical optimization contained in the Design-Expert 10.0.6.0 software was used to optimize acetin production. Usually, the software searches for a combination of parameters that satisfy the conditions set for both the parameters and responses. All the parameters and responses were set within the range limit. The desirability function of “1”, which represents the accuracy between the experimental results and the suggested values, was applied [29]. Assisted by the Design-Expert software, the second-order quadratic polynomial model, as indicated earlier in Equation (4), was used to generate several optimum reaction conditions with their corresponding responses with the desirability of “1”. Based on the suggested solutions, experiments were conducted to validate the model and the results indicated in Table 3 (only the first four solutions were reported and validated). From the results, the experimental values are close to the predicted values. This confirms the validity of the model. Reports from the various studies showed similar optimum reaction conditions with different catalysts and outcomes. The responses obtained from this study, especially the TA, which is considered the most desired products, were higher than the recently reported studies based on the conditions suggested by the software, which include temperature (126 °C), G/AA mole ratio (1:10.4), catalyst load (0.45 g), a 3 h reaction time leading to a GC of 97%, and MA, DA, and TA selectivity of 5, 28, and 67%, respectively. Kulkarni et al. reported GC of 99.1%, MA, DA, and TA selectivity of 22, 57, and 21%, respectively, and at optimum conditions of 100 °C, 1:10 G/AA mole ratio, 5 wt % catalyst, and a reaction time of 3 h using a sulfated CeO_2_–ZrO_2_ mixed-oxide catalyst. Using a composite of treated sucrose-based carbon with a silica template (SBA-15), 95% GC and selectivity of 19, 59, and 22% MA, DA, and TA, respectively, were reported at optimum conditions of 110 °C, a 1:9 G/AA mole ratio, and 0.7 g but at a higher reaction time of 6 h [22]. Okoye et al. also reported the use of sulfonated glycerol-based carbon as a catalyst with 99% GC, a selectivity of 12, 45, and 43% MA, DA, and TA, respectively, and at optimum conditions of 110 °C, a 1:3 G/AA mole ratio, 2 wt % glycerol, and a 3 h reaction time, respectively.

### 2.6. Catalyst Reusability

**Figure 14:**
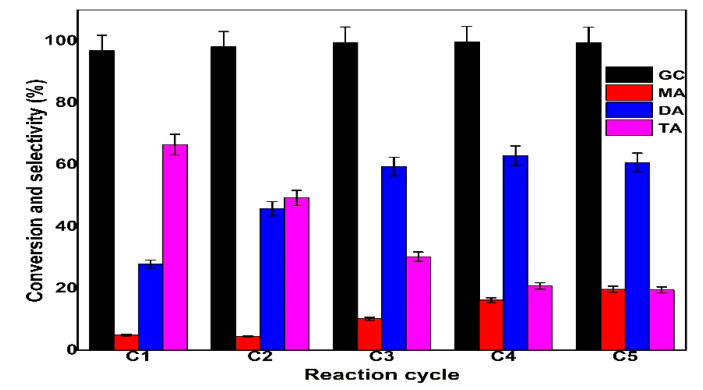
Reusability of the OMSC catalyst in glycerol conversion (GC) and selectivity to monoacetin (MA), diacetin (DA) and triacetin (TA) in five reaction cycles (C1 to C5).

**Figure 15:**
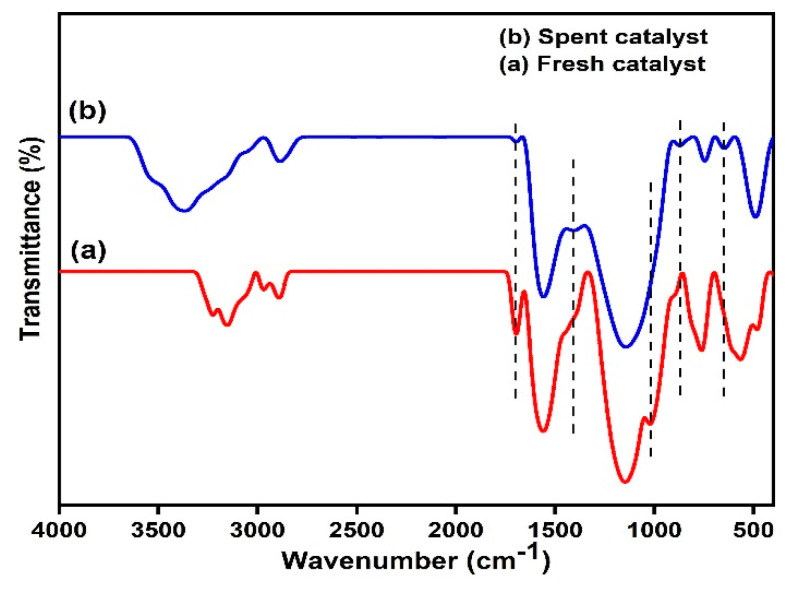
FTIR spectrum of the (a) fresh and (b) spent catalysts after the fifth cycle.

**Figure 16:**
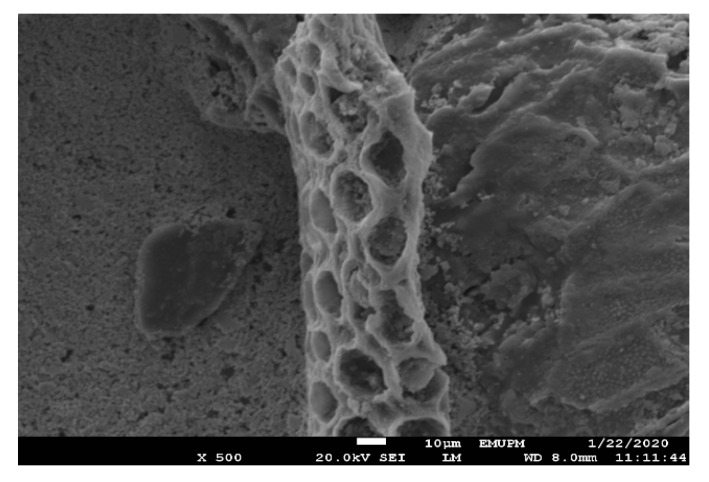
SEM image of the spent catalyst after the fifth cycle.

In heterogeneous catalysis, catalyst reusability is an important factor for its economic viability and is considered a significant advantage when compared with homogeneous catalysis [6,12,36]. Therefore, catalyst reusability in glycerol acetylation with acetic acid was conducted by consecutive batch cycles under optimal conditions. The optimum conditions of temperature (126 °C), G/AA mole ratio (1:10.4), catalyst load (0.45 g), and a 3 h reaction time were applied for the reusability study. The catalyst used after each cycle was recovered via filtration, washed with deionized water and ethanol successively, and dried in an oven at 120 °C for 2 h before being reused. The catalyst was reused for five reaction cycles. The acetin distribution was analyzed after each cycle, and the results are presented in Figure 14. After the fifth cycle, the spent catalyst was further subjected to elemental, FTIR, and SEM analysis to determine its stability or otherwise, and the results are presented in Table 4 and Figure 15; Figure 16, respectively.

The results in Figure 14 show that the glycerol conversion remains almost constant throughout the five reaction cycles (≈98%), indicating the ease of glycerol conversion to acetin. However, the selectivity of TA decreased from 66.5% in the first run to 49.2% in the second run and 30.3% in the third run. It later became stabilized in the subsequent cycles with 22.2%. Since glycerol acetylation reaction is a consecutive reaction, the decrease in TA selectivity led to an increase in DA and MA selectivity, respectively. A similar observation was reported by Keogh et al. where the glycerol conversion remained constant, but the product distribution varied slightly for the four reaction cycles, which the authors attributed to a partial loss of the catalyst during the separation process. Similarly, Goscianska and Malaika reported constant glycerol conversion for the four reaction cycles using a sulfonated sucrose–silica (SBA) composite as a catalyst. However, little variation was observed in the selectivity of the acetin, which was attributed to a small degree of leaching. The observation in our study can be attributed to the catalyst’s deactivation due to the leaching of active sites, as exhibited by the characterization results of the spent catalyst. The elemental analysis of the spent catalyst indicates a drastic drop in sulfur content after the fifth cycle with 59.3%, which implies a substantially decreased in sulfonic acid density (Table 4). A similar drastic drop in the acidity of the catalyst has been earlier reported in similar carbon-based catalysts [57,58]. The reduction in acidity in our study was buttressed by the FTIR spectrum of the spent catalyst illustrated in Figure 15. The absorption band at 1700 cm^−1^ associated with C=O reduced to a very large extend, while the absorption band around 1000 cm^−1^ attributed to S=O could not be observed in the spent catalyst (Figure 15b), except for the small band around 850 cm^−1^. The lack of appearance or reduced intensity of certain absorption bands in the spectrum of the spent catalyst, when compared to that of the fresh catalyst (Figure 15a), confirmed the leaching of the active sites. Several literature reports have indicated that deactivation of catalysts can be associated with the leaching of the active sites, which may be caused by the weak attachment of the active sites to the bulk or surface of the catalyst, hydrolysis, or attrition in the washing process [9,25,45]. The SEM image of the spent catalyst in Figure 16 does not show a remarkable difference from the fresh catalyst except for its darkness and smooth surface. This indicates that it maintained its surface morphology even after the reaction. However, the resultant TA selectivity after stabilization of the catalyst in this study is still high when compared with some reported literature [45,55].

## 3. Materials and Methods

### 3.1. Materials

All the reagents were of analytical grade and used as purchased. Sodium silicate (chemically pure), sodium hydroxide (99%), sodium chloride (99.5%), sulfuric acid (95–98%), hydrochloric acid (37%), anhydrous glycerol (99.8%), and glacial acetic acid (99.8%) were all purchased from R&M Chemicals, Ltd., Selangor, Malaysia. Acetonitrile (GC grade) and 1,3-butanediol (99.999%) were purchased from Sigma-Aldrich, Milwaukee, WI, USA. Phenolphthalein (99%) was purchased from Systerm, Selangor, Malaysia. The precursor carbon material, palm kernel shells (PKS), was obtained from a local milling industry in Selangor, Malaysia.

### 3.2. Preparation of the Mesoporous Sulfonated Carbon Catalyst

The mesoporous sulfonated carbon catalyst was prepared using the template carbonization of the PKS and subsequent sulfonation with concentrated sulfuric acid in line with our earlier work [21]. In a brief description, PKS (elemental composition: 49.6% C, 5.8 wt % H, 0.1 wt % N, and 44.5 wt % O) were washed, oven-dried, and ground. The particles with a size of ≤250 µm were collected and stored in plastic bottles for further experiments. A 200 g amount of the powdered PKS was mixed with 1 M HCl and later with dilute Na_2_SiO_3_ with adequate stirring until it hardened. The mixture was allowed to polymerize in the oven overnight and later carbonized at 800 °C under a CO_2_ environment for 4 h. After carbonization, the resultant mixture was treated with dilute NaOH with heating for 1 h, washed several times with deionized water, and dried overnight at 120 °C. The dried material was later crushed in an agate mortar and sieved again, as indicated above. A 10 g amount of the dried material was mixed with concentrated sulfuric acid (1:10 *w*/*v*) in a round-bottom flask fitted with a reflux condenser. The mixture was purged with N_2_ gas to remove any dissolved air and other gaseous impurities and later heated in an oil bath under magnetic stirring at 450 rpm. To obtain the optimized mesoporous sulfonated carbon (OMSC) catalyst, sulfonation temperature, and time, considered to be the significant factors, were varied in line with earlier studies [25,27,31]. The different sulfonation temperature and time used include 60, 90, 120, 150, and 180 °C and 1, 2, 3, 5, and 7 h, respectively. After each sulfonation process, the mixture was cooled and thoroughly washed with hot deionized water until neutral pH. The resultant sulfonated catalyst was oven-dried for 24 h at 120 °C.

### 3.3. Characterization of the Catalyst

The characterization of the OMSC catalyst was carried using various analytical methods. The sulfonic and total acid densities were estimated by the titration method, as reported in several literature articles with a slight modification [27,35,48,59]. Briefly, 0.05 g of the catalyst was mixed with a 15 mL 2 M NaCl solution and stirred at room temperature for 1 h. After that, the filtrate was titrated with 0.05 M NaOH solution using phenolphthalein indicator, and the sulfonic acid (SO_3_H) density was determined as the amount of NaOH solution consumed. In the total acid (contribution of all the acid groups) determination, 0.1 g of the catalyst was mixed with 20 mL 0.05 M NaOH solution while stirring for 1 h, and the filtrate titrated with 0.1 M HCl solution using a phenolphthalein indicator. Temperature-programmed desorption-ammonia (TPD-NH_3_) analysis was carried out on a Thermo Finnigan TPD/R/O 1100 series instrument equipped with a thermal conductivity detector (TCD) to measure the total acid site and their distribution. The elemental sulfur was determined using the CHNS analyzer (EA1112 Thermo Finnigan FLASH, Delft, Netherlands) and the sulfonic acid density also estimated from it with the assumption that all the sulfur present in the samples were due to sulfonic acid groups as reported in the literature [22,60]. Thermogravimetric analysis (TGA) was carried out with a thermal gravimetric analyzer (Mettler, Toledo 851e, Greifensee, Switzerland) equipped with differential thermogravimetric in the temperature range of 50 to 600 °C at 10 °C min^−1^ under nitrogen gas flow at 50 mL/min. The functional groups were detected using attenuated total reflectance Fourier transform infrared spectroscopy (ATR-FTIR) (Perkin Elmer 1650 Spectrometer, Waltham, MI, USA) within the scanning range of 400 to 4000 cm^−1^. X-ray diffraction (XRD) was conducted using an X-ray diffractometer (Shimadzu Model XRD 6000, Kyoto, Japan) operating with CuKα radiation with wavelength (λ) = 1.54 Å at a scanning speed of 4° min^−1^ and 2θ scan range of 10–80° at 30 kV and 30 mA. Raman spectrum was recorded using WITec Alpha 300R spectrometer (Ulm., Germany) in the range of 100–4000 nm with a laser excitation wavelength of 532 nm. The surface morphology was analyzed with SEM (JEOL, JSM-6400). The N_2_-physisorption analysis was carried out using a Micrometrics analyzer (TriStar II Plus model, Norcross, GA, USA) at −196 °C. The surface area was calculated from the N_2_ physisorption isotherm using the Brunauer–Emmett–Teller (BET) formula, while the pore size and volume were estimated using the Barrett–Joyner–Halenda (BJH) technique and the t-plot. X-ray photoelectron spectroscopy (XPS) analysis was carried out to confirm the presence of the surface functional groups using PHI Quantera II (Ulvac-PHI, Inc. Kanagawa, Japan) instrument with monochromatic Al K_α_ (hv = 1486.6 eV) as the X-ray source (0.8–50 W). The binding energies were calibrated relative to the C 1s peak at 286.2 eV obtained from the sample. The obtained data were deconvoluted into several peaks using the SmartSoft-XPS software and Multipak ECSA (Chanhassen, MN, USA).

### 3.4. Catalytic Activity

The sulfonated carbon catalysts were tested in a batch acetylation reaction system (250 mL round-bottom flask equipped with a reflux condenser and a magnetic stirrer) containing 5 g of glycerol,18.6 mL of acetic acid, and 0.5 g of the synthesized catalyst. The reaction system was conducted under reflux at 120 °C in an oil bath with magnetic stirring at 450 rpm for 3 h. At the end of the reaction, the mixture was centrifuged, filtered, and the acetin distribution quantitatively analyzed using GC-FID (Agilent 7890A) fitted with DB-Wax 30 m × 0.25 mm × 0.25 µm column using acetonitrile as solvent and 1,3-butanediol as the internal standard. A 1 µL volume of the final sample was used for the analysis and helium was used as the carrier gas at a flow rate of 1.3 mL min^−1^. The glycerol conversion (GC) and selectivity to monoacetin (MA), diacetin (DA), and triacetin (TA) were estimated using Equations (5) and (6) [45].

(5) $$\mathrm{GC}\;\left(\%\right)=\frac{\mathrm{Amount}\;\mathrm{of}\;\mathrm{Glycerol}\;\mathrm{reacted}}{\mathrm{Amount}\;\mathrm{of}\;\mathrm{Glycerol}\;\mathrm{taken}}\times 100$$

(6) $$\mathrm{Selectivity}\;\left(\%\right)=\frac{\mathrm{Amount}\;\mathrm{of}\;\mathrm{specific}\;\mathrm{product}}{\mathrm{Total}\;\mathrm{amount}\;\mathrm{of}\;\mathrm{products}}\;\times 100$$

### 3.5. Response Surface Methodology (RSM)

RSM is a collection of mathematical and statistical methods used in ascertaining the proper relation between factors and one or more responses to locate an optimum in the design space using statistical, regression modeling, and optimization techniques [11]. RSM requires that several experiments be performed to determine the direction towards the best value, repetition of the experiment for optimal factor, and, finally, construction of a mathematical model to determine the optimum conditions for validation by experiment [29].

In this study, a two-level, three-factor, face-centered central composite design (2^3^ CCD) was used to optimize the conditions of glycerol acetylation reaction over the OMSC catalyst with the view to improving triacetin selectivity. The effects of three independent variables (factors), namely temperature, glycerol-to-acetic acid (G/AA) mole ratio, and catalyst load on acetin production were investigated. The coded and actual levels of the independent variables are indicated in Table 5. In line with Equation (7), a total of 20 experiments were carried out in a randomized order as generated by the Design-Expert 10.0.6.0 software (Stat-Ease Inc., Minneapolis, MN, USA).

(7) $$N=2^{n}+2n+n_{c}$$

where *N* is the total number of runs, 2*^n^* is factorial runs, 2*^n^* is axial runs, and *n*_c_ center runs (usually in replicates). Therefore, 8 factorial points (−1 and +1), 6 axial points, and 6 replicate center points experiments were conducted. The experimental design matrix is shown in Table S1 with GC and selectivity to MA, DA, and TA set as responses (dependable variables).

The experimental data were analyzed by response surface regression using a second-order quadratic polynomial Equation (8)

(8) $$Y=\beta_{o}+\overset{3}{\underset{i=1}{\sum }}\beta_{i}x_{i}+\overset{3}{\underset{i=1}{\sum }}\beta_{ii}x_{i}^{2}+\sum^{\;}\overset{3}{\underset{i<j=1}{\sum }}\beta_{ij}x_{i}x_{j}\;$$

where *Y* is the predicted response, *β_o_*, *β_i_*, *β_ii_*_,_ and *β_ij_* are regression coefficients for the intercept, linear, quadratic, and interaction terms, respectively, while *x_i_* and *x_j_* are the coded independent variables.

The quality and significance of the model fit were evaluated using the analysis of variance (ANOVA) and coefficient of determination (R^2^), while the individual and interactive effects of the independent variables were assessed based on the surface response plots [29,53]. The numerical optimization in the Design-Expert software was used to optimize the responses by setting the desirability to 1. The obtained optimum conditions were later validated.

## 4. Conclusions

A mesoporous sulfonated carbon catalyst derived from PKS was synthesized and optimized by varying the sulfonation temperature and time. The optimal conditions of 90 °C sulfonation temperature in 5 h, resulted in a catalyst with 1.16 and 7.10 mmol/g sulfonic and total acid densities with relatively high BET surface area (217 m^2^/g). Further characterization of the resultant carbon catalyst proves that it is indeed mesoporous and amorphous in structure with good textural and thermal properties, as well as having surface oxygenated functional groups. The catalyst exhibited excellent activity in glycerol acetylation reaction with acetic acid. Upon optimizing the parameters of the reaction using RSM, results showed that a temperature of 126 °C, G/AA mole ratio of 1:10.4, and catalyst load of 0.45 g were the optimal conditions in obtaining high TA selectivity. The 66.5% TA selectivity obtained in this study was found to be the highest when compared with the recent literature reports, making it a promising catalyst. Furthermore, the reusability of the catalyst revealed efficient glycerol conversion for the five cycles (≈98%), but the selectivity to TA decreased from 66.5% in the first cycle to 22% in the fifth cycle. The characterization of the spent catalyst shows that the leaching of the most active sites was responsible for the reduction in TA selectivity.

## Figures and Tables

**Table 1:** Sulfur content and acid density of catalyst prepared at various sulfonation temperatures (5 h) and times (90 °C).

Sulfonation Temperature (°C)	S ^a^ (%)	SAD ^b^ (mmol/g)	SAD ^c^ (mmol/g)	TAD ^d^ (mmol/g)	Sulfonation time (h)	S ^a^ (%)	SAD ^b^ (mmol/g)	SAD ^c^ (mmol/g)	TAD ^d^ (mmol/g)
60	3.06	0.96	1.33	5.00	1	1.93	0.60	1.20	5.10
90	3.72	1.16	2.57	7.10	2	2.32	0.72	1.47	5.25
120	3.24	1.01	2.23	6.00	3	3.09	0.96	1.70	5.40
150	3.12	0.97	1.90	6.50	5	3.27	1.02	1.80	5.45
180	2.79	0.87	1.67	5.80	7	1.96	0.61	1.27	5.35

S = sulfur, SAD = sulfonic (SO_3_H) acid density, TAD = total acid density. ^a^ Determined using the elemental (CHNS) analysis, ^b^ calculated from the S content, ^c^ determined by titration with NaOH after ion exchange with NaCl solution, ^d^ obtained by titration with HCl after extraction with NaOH solution.

**Table 2:** Textural property and acid site density of the OMSC catalyst.

Sample	Surface Area (m^2^/g)	Pore Volume (cm^3^/g)	Average Pore Diameter (Å)	Acid Site Density ^a^ (µmol/g)
Carbonized	484	0.327	27.09	ND
OMSC Catalyst	217	0.164	47.04	9568.3

^a^ Obtained from the TPD-NH_3_ analysis, ND = not determined.

**Table 3:** The predicted and the validation of results of the numerical optimization.

Run	Conditions of Reaction			Predicted Value (%)				Desirability	Experimental Value (%)			
	T	MR	CL	GC	MA	DA	TA		GC	MA	DA	TA
1.	126	10.4	0.45	97.52	2.26	23.96	69.21	1.000 *	96.97	4.87	27.76	66.49
2.	80	12	0.2	99.47	3.11	41.95	51.93	1.000	96.87	5.68	50.03	43.07
3.	110	9	0.6	94.27	3.98	22.85	63.51	1.000	97.46	5.55	34.84	58.41
4.	110	9	0.2	98.29	3.62	37.74	57.02	1.000	97.19	5.24	39.67	54.23

* Selected by the Design-Expert software. T = temperature, MR = mole ratio of G/AA, CL = catalyst load.

**Table 4:** Sulfur content and acid properties of the fresh and the spent catalyst after the fifth use.

Sample	S ^a^ (%)	SAD ^b^ (mmol/g)	SAD ^c^ (mmol/g)	TAD ^d^ (mmol/g)
Fresh catalyst	3.72	1.16	1.57	7.10
Spent catalyst	0.95	0.30	0.33	1.69

SAD = SO_3_H acid density, TAD = total acid density. ^a^ Determined using elemental sulfur, ^b^ calculated from the S content. ^c^ determined by titration with NaOH after ion exchange with NaCl solution, ^d^ obtained by titration with HCl after extraction with NaOH solution.

**Table 5:** Independent variables (factors) with their codes and levels.

			Levels	
Factor	Unit	Code	Minimum	Maximum
			−1	1
Temperature	°C	A	80	140
Mole ratio	-	B	6	12
Catalyst load	g	C	0.2	1

## Supplementary Materials

The following are available online, Tables S1 to S5: the independent variables including their levels, the experimental design matrix and the response, as well as the ANOVA results for GC, MA, DA, and TA.
