# Supplementary material for: Optimization and Characterization of Mesoporous Sulfonated Carbon Catalyst and Its Application in Modeling and Optimization of Acetin Production
Source: Molecules. 2020 Nov 10;25(22):5221. doi: 10.3390/molecules25225221 (PMC7697787; doi:10.3390/molecules25225221)
Supplement: Supplementary file 1 [file molecules-25-05221-s001.pdf]

Supplementary Materials

Optimization and characterization of mesoporous sulfonated carbon catalyst and its application in modelling and optimization of acetin production.

Usman Idris Nda-Umar[1,2*], Irmawati Ramli[1,3,4*], Ernee Noryana Muhamad[1,3], Norsahida Azri[1,3], Yun Hin Taufiq-Yap[1,3]

[1]Department of Chemistry, Faculty of Science, Universiti Putra Malaysia, 43400 UPM Serdang, Selangor, Malaysia.

[2]Department of Chemical Sciences, Federal Polytechnic, P.M.B. 55, Bida, Niger State, Nigeria.

[3]Catalysis Science and Technology Research Centre (PutraCat), Faculty of Science, Universiti Putra Malaysia, 43400 UPM Serdang, Selangor, Malaysia.

[4]Laboratory of Processing and Product Development, Institute of Plantation Studies, Universiti Putra Malaysia, 43400 UPM Serdang, Selangor, Malaysia.

*Correspondence: uindaumar@gmail.com (U.I.N-U.), irmawati@upm.edu.my (I.R.)

Table S1: Design matrix for 2^3 CCD with the experimental response values

	Variables			Responses			
Run	Temperature (°C)	Mole ratio -	Cat. load (g)	GC (%)	MA (%)	DA (%)	TA (%)
1	110	6	0.6	87.6158	6.6523	31.7239	52.1095
2	80	9	0.6	96.4021	4.3640	25.1875	63.5204
3	110	9	0.6	94.0397	4.2684	24.1575	58.6872
4	140	12	0.2	99.5985	0.1116	31.2642	67.6047
5	110	9	0.6	94.3412	3.8696	21.0776	60.6677
6	80	6	0.2	95.0388	6.9164	39.4414	47.6165
7	80	12	0.2	99.5889	3.4120	43.5070	51.2205
8	110	9	0.2	98.5828	3.8042	34.1871	57.6553
9	110	9	0.6	94.5164	4.2426	25.9724	58.4367
10	110	12	0.6	95.8169	1.9937	20.4630	69.9515
11	140	9	0.6	96.4530	3.2362	15.9967	73.4738
12	140	6	0.2	96.2311	4.9994	32.2473	55.1466
13	110	9	1	89.9672	4.2142	14.9069	63.6361
14	80	12	1	93.4775	3.8091	15.2194	67.1568
15	140	12	1	96.1273	2.1103	9.8442	82.0707

16	110	9	0.6	93.5278	3.6061	17.0690	68.3218
17	110	9	0.6	94.6343	4.0326	24.6211	62.8543
18	110	9	0.6	94.1242	4.0634	25.6623	64.1734
19	140	6	1	84.5026	5.9163	17.9448	46.7719
20	80	6	1	83.7703	7.6660	24.5366	36.0251

GC=Glycerol conversion, MA=Monoacetin, DA=Diacetin, TA=Triacetin.

Table S2: Analysis of variance (ANOVA) for the GC

**Source**	**Sum of Squares**	**df**	**Mean Square**	**F Value**	**p-value Prob > F**	
Model	359.30	9	39.92	134.01	< 0.0001	significant
A-Temperature	2.15	1	2.15	7.21	0.0229	
B-Mole ratio	140.25	1	140.25	470.79	< 0.0001	
C-Cat load	169.70	1	169.70	569.65	< 0.0001	
AB	0.068	1	0.068	0.23	0.6443	
AC	0.59	1	0.59	1.99	0.1882	
BC	22.49	1	22.49	75.50	< 0.0001	
A^2	11.61	1	11.61	38.98	< 0.0001	
B^2	19.40	1	19.40	65.14	< 0.0001	
C^2	0.026	1	0.026	0.088	0.7727	
Residual	2.98	10	0.30			
Lack of Fit	2.19	5	0.44	2.76	0.1446	not significant
Pure Error	0.79	5	0.16			
Cor Total	362.28	19				
Std. Dev.	0.55	R-Squared		0.9918		
Mean	93.92	Adj R-Squared		0.9844		
C.V. %	0.58	Pred R-Squared		0.8995		
PRESS	36.39	Adeq Precision		43.156		

Table S3: Analysis of variance (ANOVA) for the MA selectivity

Source	Sum of Squares	df	Mean Square	F Value	p-value Prob > F	
Model	55.98	9	6.22	46.45	< 0.0001	significant
A-Temperature	9.59	1	9.59	71.64	< 0.0001	
B-Mole ratio	42.91	1	42.91	320.45	< 0.0001	
C-Cat load	2.00	1	2.00	14.94	0.0031	
AB	0.22	1	0.22	1.66	0.2269	
AC	0.39	1	0.39	2.92	0.1182	
BC	0.066	1	0.066	0.50	0.4971	
A^2	0.038	1	0.038	0.29	0.6042	
B^2	0.45	1	0.45	3.37	0.0964	
C^2	0.023	1	0.023	0.17	0.6887	
Residual	1.34	10	0.13			
Lack of Fit	1.03	5	0.21	3.36	0.1047	not significant
Pure Error	0.31	5	0.061			
Cor Total	57.32	19				

Std. Dev.	0.37	R-Squared	0.9766
Mean	4.16	Adj R-Squared	0.9556
C.V. %	8.79	Pred R-Squared	0.7852
PRESS	12.31	Adeq Precision	27.039

Table S4: Analysis of variance (ANOVA) for the DA selectivity

Source	Sum of Squares	df	Mean Square	F Value	p-value Prob > F	
Model	1341.37	9	149.04	16.70	< 0.0001	significant
A-Temperature	164.79	1	164.79	18.46	0.0016	
B-Mole ratio	65.52	1	65.52	7.34	0.0220	
C-Cat load	964.23	1	964.23	108.03	< 0.0001	
AB	1.84	1	1.84	0.21	0.6599	
AC	6.97	1	6.97	0.78	0.3975	
BC	52.53	1	52.53	5.89	0.0357	
A^2	9.73	1	9.73	1.09	0.3210	
B^2	36.04	1	36.04	4.04	0.0723	
C^2	11.82	1	11.82	1.32	0.2765	
Residual	89.26	10	8.93			
Lack of Fit	30.55	5	6.11	0.52	0.7547	not significant
Pure Error	58.71	5	11.74			
Cor Total	1430.63	19				
Std. Dev.	2.99	R-Squared		0.9376		
Mean	24.75	Adj R-Squared		0.8815		
C.V. %	12.07	Pred R-Squared		0.7311		
PRESS	384.71	Adeq Precision		16.019		

Table S5: Analysis of variance (ANOVA) for the TA selectivity

Source	Sum of Squares	df	Mean Square	F Value	p-value Prob > F	
Model	1951.30	9	216.81	18.15	< 0.0001	significant
A-Temperature	354.36	1	354.36	29.66	0.0003	
B-Mole ratio	1006.70	1	1006.70	84.25	< 0.0001	
C-Cat load	26.95	1	26.95	2.26	0.1640	
AB	21.19	1	21.19	1.77	0.2125	
AC	0.38	1	0.38	0.032	0.8618	
BC	317.12	1	317.12	26.54	0.0004	
A^2	24.77	1	24.77	2.07	0.1805	
B^2	54.84	1	54.84	4.59	0.0578	
C^2	64.70	1	64.70	5.41	0.0423	
Residual	119.48	10	11.95			
Lack of Fit	48.84	5	9.77	0.69	0.6524	not significant
Pure Error	70.65	5	14.13			
Cor Total	2070.79	19				
Std. Dev.	3.46	R-Squared		0.9423		
Mean	60.36	Adj R-Squared		0.8904		
C.V. %	5.73	Pred R-Squared		0.8148		
PRESS	383.43	Adeq Precision		18.411		
